# Considerations for Comprehensive Analyses of Sporozoite-Based Controlled Human Malaria Infection Studies

**DOI:** 10.4269/ajtmh.15-0327

**Published:** 2015-12-09

**Authors:** Andrew A. Lover

**Affiliations:** Malaria Elimination Initiative, University of California, San Francisco, San Francisco, California

## Abstract

There has been renewed interest in the use of sporozoite-based approaches for controlled human malaria infections (CHMIs), and several sets of human challenge studies have recently completed. A study undertaken in Tanzania and published in 2014 found dose dependence between 10,000 and 25,000 sporozoite doses, as well as divergent times-to-parasitemia relative to earlier studies in European volunteers, with important implications for planning future studies. Analysis of time-to-event data has had extensive development in recent years, but these methods have had limited exposure outside biostatistics. Expansion of the published analyses to include recent methodological approaches optimized for the types of data used could provide a richer analysis of these studies and may result in alternative findings. Specifically, in a re-analysis of these data using survival analysis techniques, the differences recorded in prepatent periods between the two dosing regimens do not reach statistical significance, and there is no evidence for statistically significant differences in prepatent periods between the Dutch and Tanzanian study sites. Although these findings do not impact the reported safety and tolerability of challange with cryopreserved *Plasmodium falciparum* sporozoites (PfSPZ), or invalidate the authors' hypotheses regarding naturally acquired immunity and its effect on parasite growth rates and prepatent periods, they highlight important opportunities to more fully use datasets from these trials and related CHMI experiments in the planning of future challenge studies.

Experimental injection of infective malaria parasites as sporozoites is currently undergoing a re-evaluation as a viable strategy for prevention of malaria infections in humans (using radiation-attenuated sporozoites)^1^,^2^ and for controlled human malaria infection (CHMI) (using non-attenuated sporozoites).^3^–^5^ Recently published work reports on the use of cryopreserved *Plasmodium falciparum* sporozoites (PfSPZ Challenge; Sanaria Inc., Rockville, MD) for challenge experiments in human volunteers in Tanzania.^6^

I applaud the authors' efforts to bring these critically important studies to malaria-endemic settings in sub-Saharan Africa, and agree that this is truly a milestone in global efforts toward development of platforms for CHMIs.

Although ethical and experimental aspects of human challenge experiments have received detailed consideration,^7^–^9^ the associated analytical issues have not. In this perspective, I suggest that expansion of Shekalaghe and others' analysis to include newer complementary approaches specifically optimized for time-to-event data provides a richer and more comprehensive view of these important results. Moreover, these suggestions are general, and could also be considered in other related CHMI studies with similar analytical strategies, for example.^2^,^10^,^11^

Time-to-event data (also called “survival” data, although the endpoint point may be any outcome) have several important characteristics. These data are generally skewed (non-normally distributed) and usually include censoring—that is, cases where the outcome is not known.^12^ Both of these issues present special obstacles in analysis, and the field has seen extensive growth with an increasingly large choice of analytical strategies and models. Many of these methods have the potential to augment and to complement existing approaches, and applying these methods to data from the PfSPZ studies suggests that contrary to the reported results, within the limited sample sizes, there is no evidence for statistically significant differences in dose response within the tested range of sporozoites, nor evidence for statistically significant differences in responses between the Dutch and Tanzanian cohorts to sporozoite exposure.

Analysis of the prepatent periods (time from sporozoite exposure to detectable parasites) in the original study was limited to a comparison of geometric means (GMs) via a nonparametric Wilcoxon rank-sum test. Although this strategy and related nonparametric methods are in common use in some fields, there exist several alternative methods for the analysis of time-to-event data,^13^ and a large body of literature exists with approaches that allow comprehensive analyses of these types of data.^14^ In earlier work with *Plasmodium vivax*, it was shown that application of non-survival analytical methods may lead to biased conclusions regarding dose dependence in sporozoite inoculations.^15^ In addition, although transforming the event times as GMs may address problems related to differences in dispersion, there is still the potential for biased estimates.^16^,^17^

A second issue that has received extensive attention in the epidemiological and clinical trial literature is one of potential biases from exclusion in the analysis of any patients originally randomized.^18^ The authors of the Sanaria study were unable to include all patients in their analysis as some had no recorded parasitemia, and therefore no analyzable time points; consequently, three patients were removed from the analysis. Inclusion of all patients randomized in an ‘intent-to-treat’ analysis has several advantages.^19^ First, it has the potential to avoid biased conclusions, and second, it allows use of all captured patient data, which maximizes the results of individual trials. Inclusion of all accrued patient-time from these trials is an especially important consideration in light of the inherently limited sample sizes.

Finally, the “failed” infections that were removed from the analysis may have been delayed incubation periods—although this phenomenon is better documented in *P. vivax* infections, it has also been reported in both naturally acquired *P. falciparum* infections^20^,^21^ and in other *P. falciparum* CHMI trials.^22^ While extended follow-up is neither feasible nor ethical in these challenge studies, these patients' follow-up time can still contribute to the study results using survival analysis.

There are other important challenges in analysis of these data: several of the Kaplan–Meier survival curves cross one another (see Figure 1), and the polymerase chain reaction (PCR)-based outcomes show evidence for proportional hazard violations. In these instances, assumptions underlying standard methods for survival analyses are violated (log-rank tests and Cox proportional hazard models, respectively). Although alternates for both situations have been developed (including Renyi log-rank tests^23^ and multivariate models^24^,^25^), these methods have had limited use outside of biostatistics.

Multivariate models, in general, also provide two advantages over rank-sum-type tests; they allow for adjustment for any differences in baseline covariates, and more importantly provide an estimate of effect size. This last issue is especially important, as statistical significance does not always imply a clinically or biologically important effect.^26^ Notably, two types of well-validated models that avoid limitations of the Cox model (flexible parametric survival models and regression methods using pseudovalues) are both readily implemented in many standard statistical software packages including Stata (College Station, TX), SAS (Cary, NC), and R (Foundation for Statistical Computing, Vienna, Austria).^25^,^27^–^29^

To illustrate the potential impact of complementary approaches, data from the original studies have been reanalyzed using these alternative approaches (see Figure 1

**Figure 1.**
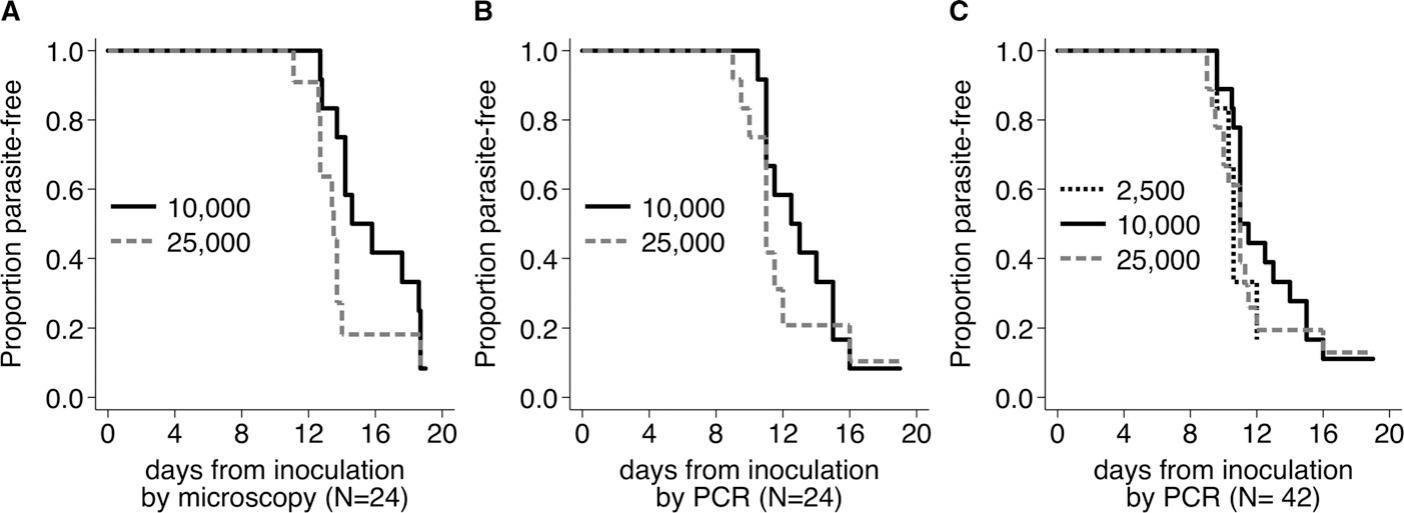
Kaplan–Meier curves comparing time-to-parasitemia by sporozoite dose groups in human sporozoite challenge studies. (**A**) Tanzanian cohort via microscopy, (**B**) Tanzanian cohort via polymerase chain reaction (PCR), (**C**) combined Tanzanian and Dutch cohorts via PCR.

and Tables 1–3). These survival analyses consisted of 1) unadjusted comparisons using log-rank tests (or alternatives where the survival curves cross) followed by 2) multivariate models.

Although the original authors' analysis of microscopy-based outcomes suggests that “Volunteers in the 10,000 PfSPZ group had a significantly different prepatent period than in the 25,000 PfSPZ group GM of 15.4 and 13.5 days, Wilcoxon, *P* = 0.023),”^6^ a survival analysis does not provide evidence of differences using a log-rank test (*P* = 0.179). Analysis of the qPCR-based end point also shows no statistically significant differences in event times (Renyi log-rank *P* = 0.266), which is consistent with the difference in GM times (11.1 and 12.6 days [*P* = 0.073], not reported in the original publication). Kaplan–Meier plots of these data are shown in Figure 1A and B. Moreover, the potential additional information from a fuller consideration of patient censoring can be seen in the differences between the full and reduced datasets (Table 1).

In addition, the original analysis found that “…the GM pre-patent period for the Tanzanians who received the 10,000 PfSPZ dosage regimen was 15.4 days, and the GM pre-patent period for the Dutch was 12.6 days (*P* = 0.0192, Wilcoxon 2-tailed).” However, in a complementary survival analysis, the unadjusted comparisons of these times (*N* = 18) with analysis as time-to-event data do not provide any evidence of statistically significant differences (log-rank *P* = 0.288; Renyi log-rank = 0.110).

Finally, to illustrate a comprehensive assessment of the overall impacts of sporozoite dosage and study site, multivariate models were used to provide covariate adjustment, and show no evidence for statistically significant differences in times-to-positivity by microscopy (Table 3) or PCR (Figure 1C, Table 2) between any of the dose categories or between the two study sites in a direct comparison of the combined cohorts (*N* = 42). Results by microscopy were modeled using a Cox proportional hazard model, whereas the PCR-based end points were examined using flexible parametric survival models due to evidence for nonproportional hazards.

Importantly, although the results from this complementary analysis do not impact the reported safety or tolerability of PfSPZ Challenge, they highlight some areas that could be considered in further analyses. It should be highlighted that these two types of analyses address subtly different questions: the original authors' analysis allows for a more ready interpretation that may be most useful for policy makers and the general public, whereas the time-to-event analysis offers a more nuanced and comprehensive, but harder to convey, set of results. Related issues have had detailed consideration in the evaluation of malaria vaccine efficacy.^30^

Secondly, these results should partially allay the authors' concerns regarding any potential differences in response to challenge between Dutch and Tanzania subjects in future work; and suggest that the Tanzanian cohort with potentially low lifetime *P. falciparum* exposure had similar responses to the malaria-naïve Dutch volunteers. However, there are likely important questions of delayed patency due to prior exposures in other populations with higher lifetime *P. falciparum* exposure that could have important implications for future work, and sample sizes should be expanded to more fully investigate these issues.

In summary, a wider selection of analytical approaches drawing on survival analysis methods could provide additional important insights to inform future work using these technically demanding and critically important experiments toward global malaria elimination.

Note: Supplemental Appendix 1 contains statistical methods; Supplemental Appendix 2 contains the dataset for this analysis.

## Supplementary Material

Supplemental Datas.

## Figures and Tables

**Table 1:** Nonparametric survival analysis comparisons between time-to-parasitemia in the 10,000 vs. 25,000 sporozoite dose groups; full data and truncated data (Tanzania study)

Source of estimates	End point	Comparison	Statistical test	Full data (*N* = 24) *P* value	Reduced data (*N* = 21) *P* value
Original publication	Parasitemia (via microscopy)	10,000 vs. 25,000 dose	Wilcoxon sign-rank	–	0.023
This work	Parasitemia (via microscopy)	10,000 vs. 25,000 dose	Log-rank	0.179	0.050*
This work	Parasitemia (via PCR)	10,000 vs. 25,000 dose	Renyi log-rank	0.266	0.102*

PCR = polymerase chain reaction.

* This is an inappropriate use of survival methods as censoring has been ignored, but is shown for completeness.

**Table 2:** Multivariate Cox model for time-to-parasitemia via microscopy in human sporozoite challenge trials, comparing dose and study cohorts (*N* = 42)

Outcome	Risk factor	HR	HR 95% CI	*P* value
Parasitemia (via microscopy)	2,500 sporozoite dose	Reference	–	–
	10,000 sporozoite dose	0.86	0.22–3.27	0.821
	25,000 sporozoite dose	1.15	0.32–4.21	0.830
Study cohort	Tanzania	Reference	–	–
	Netherlands	1.21	0.52–2.81	0.655

CI = confidence interval; HR = hazard ratio.

**Table 3:** Multivariate flexible parametric survival model for time-to-parasitemia via PCR in human sporozoite challenge trials, comparing dose and study cohorts (*N* = 42)

Outcome	Risk factor	HR	HR 95% CI	*P* value
Parasitemia (via PCR)	2,500 sporozoite dose	Reference	–	–
	10,000 sporozoite dose	0.71	0.16–3.21	0.653
	25,000 sporozoite dose	1.29	0.33–5.10	0.714
Study cohort	Tanzania	Reference	–	–
	Netherlands	1.44	0.52–3.95	0.474

CI = confidence interval; HR = hazard ratio; PCR = polymerase chain reaction.
