# Supplementary material for: Considerations for Comprehensive Analyses of Sporozoite-Based Controlled Human Malaria Infection Studies
Source: Am J Trop Med Hyg. 2015 Dec 9;93(6):1130–3. doi: 10.4269/ajtmh.15-0327 (PMC4674223; doi:10.4269/ajtmh.15-0327)
Supplement: Supplementary file 1 [file SD1.pdf]

SUPPLEMENTAL APPENDIX 1: METHODS

The end points for the survival analyses were taken from respective Tables 2 in each publication.[1,2] In the Tanzanian studies, two of the excluded patients were censored at the reported times (volunteer 40055-20 at 19 days, and volunteer 50057-20 at 11 days); insufficient data were reported to censor the third volunteer (40010-20) who was also censored on day 19. Patients in the Dutch studies were censored on day 21 when presumptive treatment was given. Dosage was modeled as an ordinal variable; inclusion as a numeric had no impact on model fit.

Model parsimony was assessed using Akaike and Bayesian information criteria, and all models were assessed for proportional hazard violations using scaled Schoenfeld residuals plus graphical comparisons log-log plots. Model fit for flexible parametric models used comparisons of overlaid plots of Kaplan–Meier curves and modeled estimates (see Supplemental Figure 1).

For situations where Kaplan–Meier curves cross, conventional log-rank type tests are invalid; therefore Renyi family tests (developed for this specific situation) were used to compare unadjusted survival between groups.[3,4]

In multivariate Cox models, proportional hazard assumptions were not met, so flexible parametric models are presented; other modeling strategies (stratified Cox, pseudovalue,[5,6] and parametric models, including Weibull, Gompertz, log-log, and lognormal) produced comparable estimates and consistent conclusions.

The Renyi tests were implemented using the survmisc package[4]; analyses were performed in Stata 13.1 (College Station, TX), and R software (version 3.0.1).[7]

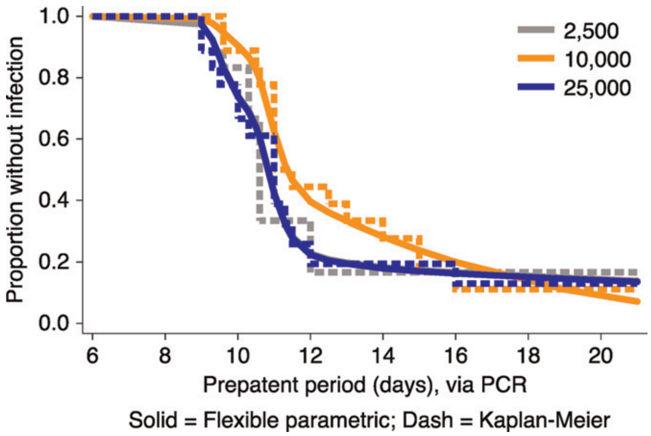

Supplemental Figure 1. Comparison of Kaplan–Meier curves and estimates from flexible parametric models, in time-to-parasitemia in controlled human malaria infections with PfSPZ Challenge.

SUPPLEMENTAL APPENDIX 2: DATA FOR ANALYSIS

Index	Study_id	PP_slide	Dose	PP_pcr	Failure	Study
1	10002-20	18.6	10,000	16	1	Tanzanian
2	10023-20	18.7	10,000	15	1	Tanzanian
3	30035-20	18.7	10,000	14	1	Tanzanian
4	40010-20	19	10,000	19	0	Tanzanian
5	50041-20	14.6	10,000	13	1	Tanzanian
6	60008-20	12.8	10,000	10.5	1	Tanzanian
7	60026-20	12.7	10,000	11.5	1	Tanzanian
8	70001-20	14.2	10,000	11	1	Tanzanian
9	70014-20	15.8	10,000	12.5	1	Tanzanian
10	70031-20	14.2	10,000	11	1	Tanzanian
11	70044-20	17.6	10,000	15	1	Tanzanian
12	90047-20	13.7	10,000	11	1	Tanzanian
13	20056-20	18.7	25,000	16	1	Tanzanian
14	20064-20	11.1	25,000	9	1	Tanzanian
15	20070-20	12.6	25,000	9.5	1	Tanzanian
16	30053-20	13.7	25,000	12	1	Tanzanian
17	30060-20	13.5	25,000	11	1	Tanzanian
18	40055-20	19	25,000	19	0	Tanzanian
19	40068-20	13.4	25,000	11	1	Tanzanian
20	50050-20	12.7	25,000	10	1	Tanzanian
21	50057-20	11	25,000	11	0	Tanzanian
22	60051-20	12.7	25,000	11	1	Tanzanian
23	60072-20	14	25,000	11.5	1	Tanzanian
24	80058-20	13.7	25,000	11	1	Tanzanian
25	696-18	12.3	2,500	9.6	1	Dutch
26	711-08	14	2,500	12	1	Dutch
27	795-06	21	2,500	21	0	Dutch
28	935-01	14	2,500	10.6	1	Dutch
29	937-20	12.3	2,500	10.6	1	Dutch
30	940-14	12.3	2,500	10.3	1	Dutch
31	119-03	12.6	10,000	9.6	1	Dutch
32	603-11	13	10,000	11	1	Dutch
33	736-04	11	10,000	9.6	1	Dutch
34	783-25	13.3	10,000	10.6	1	Dutch
35	788-21	14	10,000	11	1	Dutch
36	925-26	21	10,000	21	0	Dutch
37	647-30	14	25,000	9.3	1	Dutch
38	720-13	12.3	25,000	10.3	1	Dutch
39	789-15	21	25,000	21	0	Dutch
40	806-09	12.3	25,000	9	1	Dutch
41	909-29	14.3	25,000	11.3	1	Dutch
42	926-24	12.3	25,000	10	1	Dutch

Dose = reported sporozoite dosage; Failure = 1 = parasitemic; 0 = censored; Index = ID for analysis; PP_pcr = reported prepatent period via polymerase chain reaction (days); PP_slide = reported prepatent period via microscopy (days); study_id = patient ID in original publications; study = study cohort.

SUPPLEMENTAL REFERENCES

1. Shekalaghe S, Rutaihwa M, Billingsley PF, Chemba M, Daubenberger CA, James E, Mpina M, Juma OA, Schindler T, Huber E, Gunasekera A, Manoj A, Simon B, Savarino E, Church LWP, Hermsen CC, Sauerwein RW, Plowe CV, Venkatesan M, Sasi P, Lweno O, Mutani P, Hamad A, Mohammed A, Urassa A, Mzee T, Padilla D, Ruben A, Sim BKL, Tanner M, Abdullah S, Hoffman SL, 2014. Controlled human malaria infection of Tanzanians by intradermal injection of aseptic, purified, cryopreserved *Plasmodium falciparum* sporozoites. *Am J Trop Med Hyg 91:* 471–480.
2. Roestenberg M, Bijker EM, Sim BKL, Billingsley PF, James ER, Bastiaens GJH, Teirlinck AC, Scholzen A, Teelen K, Arens T, van der Ven AJAM, Gunasekera A, Chakravarty S, Velmurugan S, Hermsen CC, Sauerwein RW, Hoffman SL, 2013. Controlled human malaria infections by intradermal injection of cryopreserved *Plasmodium falciparum* sporozoites. *Am J Trop Med Hyg 88:* 5–13.
3. Klein JP, Moeschberger ML, 2005. *Survival Analysis: Techniques for Censored and Truncated Data, Vol.*, 2nd edition. New York, NY: Springer.
4. Dardis C, 2015. *survMisc: Miscellaneous Functions for Survival Data*. Available at: http://cran.r-project.org/web/packages/survMisc/index.html. Accessed February 5, 2015.
5. Andersen PK, Perme MP, 2010. Pseudo-observations in survival analysis. *Stat Methods Med Res 19:* 71–99.
6. Parner ET, Andersen PK, 2010. Regression analysis of censored data using pseudo-observations. *Stata J 10:* 408–422.
7. R Core Team, 2013. R: A Language and Environment for Statistical Computing. Vienna, Austria: Foundation for Statistical Computing. Available at: http://www.R-project.org/.
